# The largest epicenter of the coronavirus outbreak in Vietnam

**DOI:** 10.1017/ice.2020.128

**Published:** 2020-04-13

**Authors:** Trang H.D. Nguyen, Danh C. Vu

**Affiliations:** 1 Institute of Biotechnology and Food Technology, Industrial University of Ho Chi Minh City, Vietnam; 2 Food for Health Center, University of Nebraska, Lincoln, Nebraska, United States; 3 Faculty of Technology, Van Lang University, Ho Chi Minh City, Vietnam

*To the Editor—*As of April 1, the total number of SARS-Cov-2–positive cases in Vietnam reached 218, and 37 of these were infected within a public hospital in Hanoi, the capital of Vietnam.^1^ Thus far, this hospital is the largest COVID-19 hotspot in the country.

**Fig. 1.**
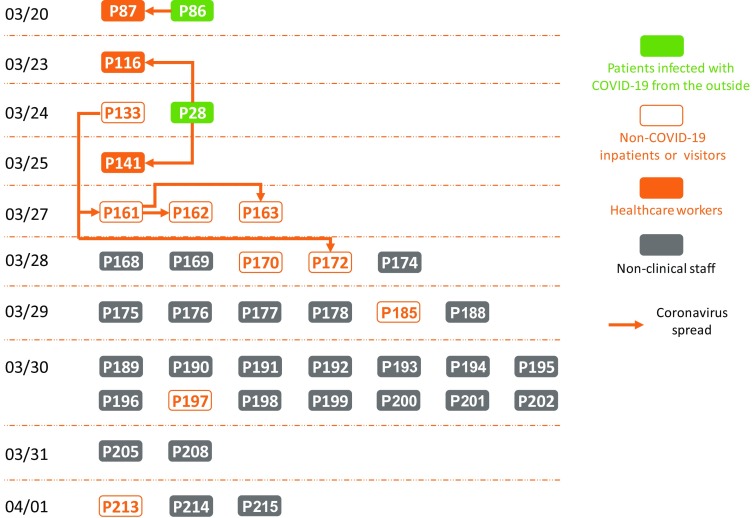
The chronology of the confirmed cases of COVID-19 in the public hospital where the first healthcare associated COVID-19 infections occurred (as of April 1, 2020). Each rectangle represents a patient numbered by the Vietnam Ministry of Health.

Three patterns of transmission occurred in the hospital: (1) between healthcare workers (HCWs), (2) from COVID-19 patients to HCWs, and (3) from nonclinical hospital staff to others. Figure 1 illustrates a timeline of the spread of the SARS-Cov-2 virus within the hospital from the first confirmed case on March 20 to the most recent case on April 1. The first SARS-Cov-2–positive case was a medical worker (P87), who was in close contact with a SARS-Cov-2–infected nurse (P86).^2^ With the exception of the 2 infected cases, P86 and P28, who contracted the coronavirus from the outside, no HCW, non–COVID-19 inpatients, or visitors had tested positive for the coronavirus. Since March 28, SARS-CoV-2 infections among nonclinical staff have emerged, and subsequently, 25 of the 37 COVID-19 cases (68%) were nonclinical staff working in the dining hall of the hospital. These catering workers were responsible for preparing meals and delivering food and hot water to patients and visitors across the hospital daily, and it is likely that these nonclinical staff are the main contributors to the spread of the virus within the hospital. Healthcare-associated infection is known characteristic of coronavirus-related diseases and a leading route of transmission.^3^

Because the confirmed cases linked to healthcare-acquired infection have only very recently been revealed and are increasing in Vietnam, several measures have been implemented to stop the spread of the disease in this hospital^4^:

1. The admission of new patients has been suspended in this hospital. Approximately 5,000 employees and non-COVID-19 inpatients of the hospital are being tested for coronavirus. Current patients are discharged only allowed if they test negative.
2. Quick COVID-19 tests have been conducted for those having close contacts with the nonclinical staff and residents living nearby the hospital.
3. Immediate lockdown has been imposed on the hospital units involved in the COVID-19 spread, and as of March 28, on the entire facility. Disinfection was conducted at the hospital in the evening of the same day.

Based on the transmission dynamics described earlier, we conclude that the major contributing factor to the significant increase in healthcare-acquired infections is extensive access of nonclinical staff across the hospital. These nonclinical workers did not properly implement effective personal protection and lacked sufficient preparedness for the emerging infectious disease during their work shifts, leading to cross contamination between the hospital units.

After the implementation of lockdown at the hospital and other mitigation measures, the numbers of new confirmed cases reported on March 31 and April 1 have begun to decrease. However, this decrease does not indicate that the outbreak in the hospital has been successfully eliminated. Therefore, in addition to the measures above, the hospital should take into consideration the following suggestions to stave off widespread coronavirus transmission in this healthcare facility and in the community:

1. Makeshift healthcare facilities are needed to accommodate non–COVID-19 outpatients and visitors as well as HCWs.
2. Because evidence increasingly favors the use of face masks, nonclinical staff should strictly wear new face masks and gloves and should update their daily health status using COVID-19 monitoring apps, such as NCOVI software developed for Vietnamese citizens.
3. Transparency in healthcare leaders’ response to the COVID-19 infections should be prioritized for the COVID-19 illness.
4. A targeted educational strategy to improve COVID-19 health literacy and knowledge for nonclinical staff in hospitals should be urgently considered.

We offer our experience with the COVID-19 outbreak and subsequent recommendations to benefit other hospitals and institutions similarly fighting this disease worldwide.
